# A Preterm Infant With Gestational Age of 21^+4^ Weeks

**DOI:** 10.1002/ccr3.71119

**Published:** 2025-10-09

**Authors:** Haifeng Zong, Bingchun Lin, Yingsui Huang, Shan Jiang, Yurong Yuan, Xiaoyuan Xiong, Zhichun Feng, Chuanzhong Yang

**Affiliations:** ^1^ Neonatal Intensive Care Unit, Shenzhen Maternity and Child Healthcare Hospital, Women and Children's Medical Center Southern Medical University Shenzhen China; ^2^ Institute of Pediatrics The Seventh Medical Center of PLA General Hospital Beijing China

**Keywords:** extremely low birth weight, extremely preterm, newborn, premature

## Abstract

We report a case of a male infant who was conceived through in vitro fertilization, with a gestational age of 21^+4^ weeks. The infant was discharged home at 42 weeks' postmenstrual age without significant cranial morbidities.

## 1 Introduction

Periviable gestation, as the gestational age (GA) spectrum, is accompanied by severe morbidity and mortality rates in survivors at the lower threshold. GA of 22 weeks has been recognized as a lower threshold of periviable gestation, while such infants are potential candidates for selective resuscitation [1]. Survivors under the periviable GA are rare, with just sporadic reports [2, 3, 4]. In this study, one preterm infant with a GA of 21^+4^ weeks was reported. He is the youngest survivor so far in China, and still survives with no significant cranial morbidities. The follow‐up results show that the infant has a good prognosis.

## 2 Case History/Examination

A male infant 21^+4^ weeks in GA was born at Shenzhen Maternity & Child Healthcare Hospital (Shenzhen, China) in 2023. His mother was 29 years old, gravida 1 para 0, and healthy with no diabetes or hypertension. Prenatal tests revealed uterine myoma and colpomycosis before delivery. Following in vitro fertilization, she was pregnant following marriage for 6 years. She carried out regular prenatal checks with no special medical problems. GA was defined as the duration from the day of oocyte retrieval, plus 14 days [5].

At the GA of 20^+5^ weeks, due to a cervical length of 1.4 cm and cervical funneling, she was transferred to Shenzhen Maternity & Child Healthcare Hospital in emergency and received ritodrine and magnesium sulfate. Clinical chorioamnionitis was not diagnosed; later, placental biopsy verified the absence of chorioamnionitis. One neonatologist and one obstetrician were responsible for prenatal parental counseling. Both parents were informed of the rarity of resuscitation for an infant 21 weeks in GA and the lack of previous experience of the medical team from Shenzhen Maternity & Child Healthcare Hospital with these infants. There were just 5 successful cases in infants at 22 weeks in GA. Therefore, a favorable outcome for this infant was difficult. Nonetheless, his parents expressed the strong wish for full resuscitation and any effort possible; therefore, antenatal dexamethasone was prescribed for a complete course to perform impending delivery (intramuscular administration of dexamethasone at 6 mg for 4 times at 12‐h intervals). Six days later, at the GA of 21^+4^ weeks, regular contractions appeared, and spontaneous induction of vaginal delivery was completed, as assisted by the neonatal resuscitation team.

## 3 Differential Diagnosis, Investigation, and Treatment

The baby's heart rate was 50 beats/min without respiratory effort after birth, and he was intubated with a 2.0‐mm endotracheal tube within 30 s after birth, and invasive positive pressure ventilation was initiated. Pulmonary surfactant was administered immediately via endotracheal tube following the first breath in the delivery room. His heart rate became > 100 beats/min following receiving positive pressure ventilation via tracheal tube for 1 min, and the 1, 5, and 10‐min Apgar scores were 6, 7, and 8, respectively. His birth weight was 450 g, length was 26 cm, and head circumference was 18 cm. Through resuscitation within the delivery room, delayed umbilical cord clamping and umbilical cord milking were not performed. Umbilical arterial catheter (UAC) and umbilical venous catheter (UVC) were inserted immediately after admission into the Neonatal Intensive Care Unit (NICU). After UVC insertion, antibiotics and prophylactic antifungal agents were administered. Moreover, the infant was supported with patient‐triggered mechanical ventilation under the synchronized intermittent mandatory ventilation (SIMV) mode. A sedative agent was not used. Total parenteral nutrition was given on the first day after admission. The total fluid intake was initiated at 90 mL/kg/day on the first day, which was later elevated to 165 mL/kg/day on the 7th day. After delivery, the incubator temperature was 37.2°C while the humidity was 95%.

With regard to respiratory management, invasive respiratory support was conducted in the SIMV + pressure support ventilation + volume guarantee mode. At 28^+6^ weeks of postmenstrual age (PMA), the infant was extubated to noninvasive respiratory support, including nasal continuous positive airway pressure and nasal intermittent positive pressure ventilation. At 35^+6^ weeks of PMA, the infant was weaned to high/low‐flow nasal cannula. At 39^+5^ weeks of PMA, the infant weaned off oxygen therapy. Moreover, two courses of postnatal dexamethasone were used on the 22nd and 38th days (DART regimen). Bronchopulmonary dysplasia was grade II according to the Jensen criteria [6].

**FIGURE 1:**
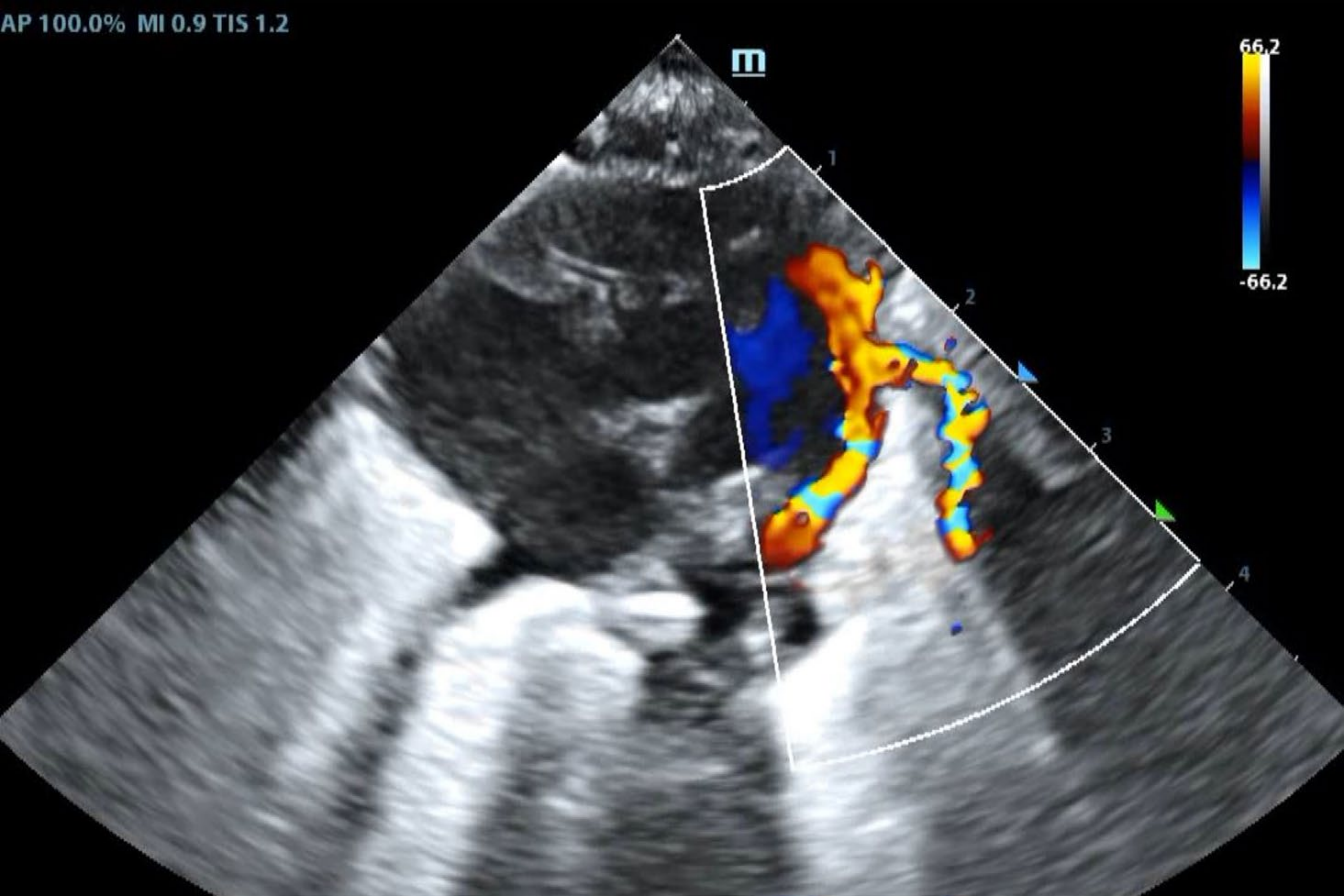
Echocardiography performed 1 week after birth revealed a patent ductus arteriosus, which was managed conservatively with no surgical or pharmacological management.

Regarding circulatory management, UAC and UVC were inserted. The umbilical catheters were eliminated in the 2nd week and substituted with peripheral lines. After circulatory status was evaluated using echocardiography and invasive artery blood pressure, cardiovascular drugs, like dopamine, epinephrine, and dobutamine were not used during the hospital stay. Hydrocortisone was administered in the first 9 days (0.25 mg/kg, q6h × 3 day, q8h × 2 day, q12h × 2 day, qd × 2 day). On day 1, the invasive arterial blood pressure was 34–39/22–28 (26–32) mmHg, and those on days 2, 3, 4, 5, 6, and 7 were 34–39/22–29 (25–31) mmHg, 34–40/23–29 (25–32) mmHg, 34–40/21–24 (25–31) mmHg, 34–42/21–25 (25–31) mmHg, 35–41/18–23 (24–29) mmHg, and 36–43/18–23 (24–28) mmHg, respectively. Serial echocardiography revealed a patent ductus arteriosus (PDA) of 0.1–0.16 cm (Figure 1). Strict long‐term fluid restriction was not applied. According to our institutional policy, PDA was managed conservatively with no surgical or pharmacological management. Echocardiograms revealed that PDA was close to closure before discharge.

On day 2, enteral feeding was initiated with donor breastmilk via the nasogastric tube at 0.5 mL/3 h for 6 days. In addition, the enteral feeding persisted by gradually and incrementally increasing the volume till the infant could tolerate full feeding, finally reaching 120 mL/kg/day at 27^+4^ weeks of PMA, without experiencing necrotizing enterocolitis. At last, gastric tube removal was completed at 37^+4^ weeks of PMA, with successful full oral feeding being achieved. The exclusive fortified breastfeeding was maintained during his hospital stay. The body weight measured at 40 weeks of PMA was 3260 g, the length and head circumference were 46 and 33 cm separately.

The serial cranial ultrasound scans and brain magnetic resonance imaging at 40 weeks of PMA revealed no detectable abnormalities, and myelination was equivalent to that in a normal term infant. Phenobarbital was not administered for sedation in the early days after birth. Newborn hearing screening was conducted, and the infant passed the screening. Brainstem auditory evoked potential examination revealed no obvious abnormality at 42 weeks of PMA.

To prevent infections, antibiotics were initiated at birth and continued until week 2. The infant had 3 episodes of blood culture‐negative sepsis at days 43, 70, and 90. The infections resolved after 1–2 weeks of antibiotic treatment. Probiotics were used for a short time after antibiotic treatment to improve intestinal dysbacteriosis.

Retinopathy of prematurity was screened regularly, and the results showed bilateral zone II, stage 3 plus at 34 weeks of PMA, and this was managed through intraocular ranibizumab administration (Lucentis; Genentech, South San Francisco, CA).

Suspended red blood cells were transfused at days 11, 25, 46, and 74. The restrictive transfusion regimen was applied, and the threshold of red blood cell transfusion was the hematocrit of 25%. Erythropoietin was applied to prevent anemia since 28 weeks of PMA.

## 4 Conclusion and Results (Outcome and Follow‐Up)

At 42 weeks of PMA, the infant was discharged with no gavage tube or supplemental oxygen. His body weight was 4000 g (50th percentile), length was 48 cm (< 10th percentile), while head circumference was 34 cm (10–50th percentile). The baby is now 9 months of corrected age. He began to lift his chest off the table at 2 months. By 4 months of corrected age, he could turn over, reach for objects, put them in his mouth, interact with others, and laugh out loud. By 6 months of corrected age, he could sit with his hands propped in front of him. By 9 months of corrected age, he can be pulled to stand and creep. He can play with toys from a seated position, hold food to take bites, and respond to simple commands.

It is worth noting that bedside ultrasound has a key effect on managing this infant, including the use of PS during resuscitation, UAC and UVC positioning, PDA management, fluid and electrolyte management, vasoactive medication, position management, ventilator parameter setting, ventilator weaning, and postnatal corticosteroid usage. In our center, bedside ultrasound is routinely used as an auxiliary tool for treatment and care, largely replacing X‐rays. This not only significantly improves the treatment outcome but also greatly reduces the X‐ray exposure and reduces the hospitalization cost. For the baby, his 143‐day hospitalization costs were only $51,245 (CNY370,000).

## 5 Discussion

The present report documented the rarity of this infant born below the lower threshold of viability. Patient management must be personalized according to risk factors and the parents' wishes. Notably, accurate estimation of GA is crucial. In this case, conception took place through in vitro fertilization; therefore, GA was verified accurately as 21^+4^ weeks. A lot of risk factors are related to unfavorable outcomes. In spite of the above risks, this infant did not have any significant cranial morbidities.

A dilemma may exist regarding the feasibility of resuscitation for infants with a GA of 21 weeks, and the possibility of infant survival post‐birth. In 2015, periviable birth was defined as 22^+0^ to 25^+6^ weeks of GA. At present, Obstetric and Pediatric Associations do not recommend attempting resuscitation before 22 weeks of GA [7, 8]. Data regarding outcomes for babies with a GA of 21 weeks are lacking, while active intervention with a GA of 22 weeks remarkably enhances infant outcomes [9]. Since 2021, our center has saved 5 cases with a GA of 22 weeks, and the follow‐up results show that all of them have a good prognosis. This case is the first preterm infant with a GA of 21 weeks saved at our center, and is also the youngest surviving preterm infant in China so far. Therefore, such benefits may extend to the 21 weeks of GA. Actually, one successful case is not sufficient to advise active obstetric and neonatal interventions for additional 21‐week pregnancies. The obstetric and neonatal team should tell the pregnant woman the predicted outcomes associated with either aggressive or non‐aggressive obstetric intervention for their baby with a GA of 21 weeks. After providing active medical care to the case, infants with a GA of 21 weeks are unnecessarily associated with death, regardless of its high probability. It should be recognized that reliable data for guiding clinical decision‐making are scarce. It is necessary to present such data comprehensively, thus fulfilling the autonomy‐based obligation to both pregnant women and their families.

Decision‐making on resuscitation for babies with the GA of 22 and 23 weeks can be complicated and greatly variable among institutions [9, 10]. The care for such babies can be regulated in different ways based on society and culture. Certain countries have set a distinct line between starting or suppressing intensive care, whereas others allow parents to take part in decision‐making [11]. In addition, more data about the outcomes of such infants are necessary for making optimal treatment decisions. As recommended by the American Academy of Pediatrics, decision‐making regarding resuscitation for babies with the GA of 22–24 weeks must be personalized and family‐centered, based on parental values and maternal beliefs [8]. According to Chinese regulations, infants with a GA of 22–23 weeks can be actively resuscitated with the informed consent of their parents [12]. Government‐provided insurance covers most medical costs. We have actively resuscitated 35 infants with the GA ≤ 23^+6^ weeks from 2022 to 2023 in our center, of which 23 (65.7%) survived. Infants with the GA of 22 weeks had a survival rate of 31.3% (5/16) in 2021–2023. We also followed up 13 infants with the GA ≤ 23^+6^ weeks discharged before 2021, and neurodevelopmental impairment occurred in only 3 infants. In the United States, the survival rate of infants with the GA of 23 weeks increased from 24.4% in 2008–2011 to 50.8% in 2018–2020, while that in those with the GA of 22 weeks increased from < 5% in 2008–2011 to 27.2% in 2018–2020 [13, 14, 15]. According to one national survey in Japan, aggressive resuscitation in newborns with a GA of 22 weeks was carried out in 81% of NICUs, including 42% of NICUs receiving resuscitation, despite their parents' wishes [16]. An observational study retrospectively examined 29 infants with the GA of 22 weeks receiving active resuscitation in Japan, with a survival rate of 82.8% (24/29) [17]. Extremely premature neonates are called “periviable” regardless of progress in neonatal medicine, which can be associated with great mortality and morbidity rates [18]. A meta‐analysis on proactive treatment with the GA of 22 weeks reported a total survival rate of 29.0% [95% confidence interval (CI), 17.2–41.6]. Meanwhile, the overall survival rate with no major and moderate/severe morbidities was 11.0% (95% CI, 8.0–14.3) and 37.0% (95% CI, 14.6–61.5), respectively [19]. In China, for disabled infants, the government has been providing subsidies for their families; if needed, there are special institutions for these infants with disabilities, and there are also many funds available to support these babies and their families. During the management of this infant, like the American Academy of Pediatrics‐recommended method [8], we first counseled the mother against resuscitation. However, both parents were subfertile, and it took them 6 years to conceive this baby. They had a firm choice although they were informed of the great mortality rate and serious neurodevelopmental sequelae. Consequently, we initiated the resuscitation of this infant and then proceeded to full life‐sustaining treatment.

Collectively, the infant born at 21^+4^ weeks of GA survives at present with a good outcome. This study demonstrates the good survival and intervention for a periviable preterm neonate with a GA of 21 weeks. More investigation is warranted to clarify the best treatment for such periviable neonates. Although this case is not sufficient to advise active obstetric and neonatal interventions for 21‐week pregnancies, it shows the progress of individualized management in these “periviable” infants.

## Author Contributions

**Haifeng Zong:** conceptualization, funding acquisition, project administration, writing – original draft. **Bingchun Lin:** conceptualization, funding acquisition, supervision, validation, visualization, writing – review and editing. **Yingsui Huang:** data curation, formal analysis, investigation, methodology, project administration, writing – review and editing. **Shan Jiang:** data curation, formal analysis, investigation, methodology, project administration, writing – review and editing. **Yurong Yuan:** data curation, formal analysis, investigation, methodology, project administration, writing – review and editing. **Xiaoyuan Xiong:** data curation, formal analysis, investigation, methodology, project administration, writing – review and editing. **Zhichun Feng:** conceptualization, funding acquisition, supervision, validation, visualization, writing – review and editing. **Chuanzhong Yang:** conceptualization, funding acquisition, supervision, validation, visualization, writing – review and editing.

## Ethics Statement

This study was performed in line with the principles of the Declaration of Helsinki. The study was approved by the ethics committee of the hospital (No. SFYLJ2023048). The parents of the patients provided written informed consent for publication of this study.

## Consent

The study was approved by the Ethics Committee of the Shenzhen Maternity & Child Healthcare Hospital, and written informed consent was obtained from the parents.

## Conflicts of Interest

The authors declare no conflicts of interest.

## Data Availability

The datasets generated during and/or analyzed during the current study are available from the corresponding author on reasonable request.
